# Systems-level reconstruction of kinase phosphosignaling networks regulating endothelial barrier integrity using temporal data

**DOI:** 10.1101/2024.08.01.606198

**Published:** 2024-08-05

**Authors:** Ling Wei, Fred D. Mast, John D. Aitchison, Alexis Kaushansky

**Affiliations:** 1 Center for Global Infectious Disease Research, Seattle Children’s Research Institute, Seattle, WA 98109, United States; 2 Department of Pediatrics, University of Washington, Seattle, WA 98105, United States; 3 Department of Biochemistry, University of Washington, Seattle, WA 98105, United States; 4 Department of Global Health, University of Washington, Seattle, WA 98105, United States

**Keywords:** endothelial barrier permeability, thrombin, phosphosignaling network, Temporal Pathway Synthesizer (TPS), Temporally REsolved KInase Network Generation (TREKING)

## Abstract

Phosphosignaling networks control cellular processes. We built kinase-mediated regulatory networks elicited by thrombin stimulation of brain endothelial cells using two computational strategies: Temporal Pathway Synthesizer (TPS), which uses phosphoproetiomics data as input, and Temporally REsolved KInase Network Generation (TREKING), which uses kinase inhibitor screens. TPS and TREKING predicted overlapping barrier-regulatory kinases connected with unique network topology. Each strategy effectively describes regulatory signaling networks and is broadly applicable across biological systems.

Protein kinases regulate numerous cellular processes through phosphorylation of target proteins. These series of phosphosignaling events are triggered in response to environmental changes and propagate within a network structure. The function of kinases in cell signaling and regulation of cellular phenotypes has been intensively explored in different contexts, including in mediating brain endothelial barrier integrity ^1^. The integrity of the blood-brain barrier (BBB) is essential for maintaining the homeostasis of the central nervous system (CNS) ^2^. Permeability of brain endothelium is tightly controlled by cell-cell junctions and focal adhesions, whose functions are heavily regulated by kinases ^1–3^. Therefore, understanding the network-level connectivity of these barrier-regulatory kinases and their temporal dynamics is critical for developing targeted therapeutic strategies for treating vascular leakage.

In recent years, computational tools and modeling approaches have been advanced to facilitate reconstructing high-confident, systems-level protein signaling networks based on proteomics data and prior knowledgebases, but they often fall short of providing detailed mechanistic understanding and/or fine temporal information on cell signaling and their function in mediating cellular phenotypes. For example, Babur *et al.* developed a pathway extraction method that maps high-throughput proteomics data to the Pathway Commons database. They used a graphical pattern search framework to identify the protein-protein interactions (PPIs) that explain correlated changes in proteomics profiles ^4^. Network inference methods like dynamic Bayesian networks have been implemented on time course phosphorylation data to infer protein signaling network structure specific to biological context without relying on user-defined parameters ^5–7^. Ordinary differential equation (ODE) models are kinetic models that have been conventionally applied to analyze biochemical reaction networks, which can capture the mechanistic details of biochemical processes and predict the temporal evolution of variables that cannot be directly measured ^8^. Unfortunately, building robust medium- or large-scale ODE models is challenging due to lack of detailed mechanistic description of the biological system or sufficient experimental data for parameterization ^8,9^. Unbiased network reconstruction has benefitted from the development of logic modeling, which employs either discrete logic models or continuous ODEs transformed from discrete logic models, followed by optimization to fit experimental data, to generate predictive models that describe context-specific signaling within the generic protein signaling networks ^10,11^.

Besides building signaling networks from phosphoproteomics data, which are typically collected at relatively sparse time points, network generation pipelines can use functional screens with fine temporal resolution as input along with known PPIs ^12,13^. For example, Bello *et al.* extended the kinase regression (KiR) approach that was initially designed to identify key kinase regulators of a particular cellular phenotype from quantitative drug screens. Their KiRNet method maps KiR hits onto existing PPI networks using network propagation to create kinase-centered, functional network models associated with the phenotype of interest ^12^. We recently developed a novel approach called TREKING that integrates temporal drug screen data with a known kinase-substrate phosphorylation database to infer functional kinase signaling networks with temporal resolution ^13^.

Köksal *et al.* have also developed a network inference algorithm specifically to model time course data ^14^. Their Temporal Pathway Synthesizer (TPS) uses time-resolved phosphoproteomics data as input ^14^. TPS assembles signaling pathways by first extracting a subnetwork that connects the phosphorylated proteins to the source node(s) from a background PPI network. It then uses the temporal phosphorylation data to systematically examine all directed, signed edges in the subnetwork to determine how signals propagate from source node(s). Finally, TPS consolidates all valid paths into a unified network that represents the pathway structures facilitating phosphosignal propagation.

Using phosphoproteomics data to build kinase signaling networks offers a distinct perspective compared to networks derived from functional assays. Phosphoproteomics help addressing how phosphosignals propagate within phosphosignaling networks, while functional assays can relate cellular phenotypes to functional kinases. Comparative analysis between these two network reconstruction approaches will be insightful for interpreting signaling events crucial to the phenotype of interest.

Here, we reconstructed a phosphosignaling network regulating thrombin-induced endothelial barrier disruption and recovery using the TPS algorithm (Fig. 1). We then compared this network with the TREKING network we built previously using kinase inhibitor screens in the same cellular background. The input time series data for TPS were collected from western blot experiments as described in ^13^. Briefly, human brain microvascular endothelial cells (HBMECs) were treated with thrombin and phosphorylation status on activation sites of 25 kinases and 3 non-kinase proteins known to regulate barrier function were collected at nine time points across a 6-hour time window after thrombin treatment. Data on 17 kinases have been published previously ^13^, and data on the remaining 8 kinases and 3 non-kinase proteins generated in this study is available in Table S1. The input network that connects phosphorated proteins to the source node, proteinase-activated receptor 1 (F2R/PAR1), was generated using Omics Integrator ^17,18^ (Table S2). The input files for TPS were prepared according to ^14^ and are described briefly in Methods.

The network generated with TPS resulted in 966 directed edges, of which only five edges are signed, primarily due to the sparsity of the input time series data (Fig. 2a, Table S3). The model predicts that both ABL proto-oncogene 1 non-receptor tyrosine kinase (ABL1/c-Abl) and mitogen-activated protein kinase 8 (MAPK8/JNK1) activate paxillin (PXN), consistent with previous findings ^19,20^. However, the model’s prediction that protein tyrosine kinase 2 (PTK2/FAK) inhibits PXN contradicts established literature ^21^ (Fig. 2a). To make comparisons with the TREKING network, the current version of which includes only kinase-to-kinase connections, we extracted the kinase-kinase interactions from the TPS network, resulting in a subnetwork of 153 edges (Fig. 2b (bottom left), Table S4). Among the 126 kinases predicted by TREKING, 56 (70%) kinases were also inferred by the TPS model, demonstrating the ability of these two distinct methodologies to infer the set of kinases activated and play a functional role in barrier regulation. Multiple members of the canonical MAPK cascades were predicted by both TPS and TREKING, including extracellular signal-regulated kinases (ERKs; MAPK1/ERK2 and MAPK3/ERK1), Jun N-terminal kinases (JNKs; MAPK8/JNK1 and MAPK9/JNK2) and p38 MAPKs (MAPK14/p38α), whose roles in temporal barrier function have been characterized ^13^. Additionally, one of the switch kinases, MAPK activated protein kinase 2 (MAPKAPK2/MK2), identified by TREKING as having both barrier-disruptive and barrier-restorative roles, was also predicted by TPS to mediate barrier properties, though TPS does not distinguish between barrier-disruptive or barrier-protective functions.

Among the 153 directed kinase-to-kinase connections in the TPS network, 50 (32.7%) are also present in the TREKING network (Fig. 2b, bottom). Both TPS and TREKING predicted that regulation of MAPKAPK2/MK2 by MAPK1/ERK2 and MAPK14/p38α is present when endothelial cells are treated with thrombin (Fig. 2c). TREKING predicted that MAPK1/ERK2-MAPKAPK2/MK2 interaction is crucial for barrier disruption, while MAPK14/p38α-MAPKAPK2/MK2 interaction is important for late barrier recovery. TPS does not provide this type of information because it does not incorporate phenotypic information for network reconstruction. TPS also inferred 103 kinase-to-kinase connections not captured by TREKING, including several signaling hubs such as outgoing signals from protein kinase cAMP-activated catalytic subunit alpha (PRKACA), SRC and protein kinase C alpha (PRKCA), and incoming signals to glycogen synthase kinase 3 beta (GSK3B) (Fig. 2c).

TPS’s ability to infer interactions between kinase regulators and their non-kinase substrates makes it a powerful tool for linking upstream kinase signaling to cellular phenotypes, as proteins directly associated with phenotypes are often better characterized and usually non-kinase. In this study, the TPS network predicted that cadherin 5 (CDH5), an endothelial adherens junction protein, is regulated by SRC. It also predicted that heat shock protein family B member 1 (HSPB1/HSP27), which directly regulates actin organization in endothelial cells ^22^, is regulated by MAPK kinases MAPK14/p38α and MAPKAPK2/MK2, PRKACA, and protein kinase CGMP-dependent 1 (PRKG1) (Table S3). These interactions in barrier regulation have been experimentally validated ^22–24^. Additionally, TPS predicted that paxillin (PXN), a cytoskeletal protein involved in actin-membrane attachment at focal adhesions, is regulated by Src family kinases FYN and SRC, p21 (RAC1) activated kinase 1 (PAK1), and several MAPK kinases, including MAPK1/ERK2, MAPK3/ERK1, MAPK8/JNK1 and MAPK14/p38α (Table S3). These regulatory interactions on paxillin (PXN) and their function in barrier regulation are also supported by previous studies ^25–27^.

In addition to providing a summary network, the TPS pipeline also outputs the activity windows of each node in the network. However, due to the sparse sampling of the input time series data, TPS was unable to identify precise activity types (activation, inhibition, inactive) for proteins at any given time point, except for those with available time course data. This highlights a limitation of the TPS methodology in reliably inferring global signaling cascades with small input datasets.

In this study, we compared two computational approaches, TPS and TREKING, in reconstructing kinase signaling networks describing phosphosignal propagation within brain endothelial cells after thrombin stimulation. Overall, TPS and TREKING provide different insights on kinase regulatory networks associated with cellular phenotypes. TPS focuses on the activity of functional proteins, while TREKING focuses on the functionality of kinases altering phenotypes. Since the phosphorylation state of a kinase does not directly imply its function, which is also impacted by its subcellular localization and presence of substrates, discrepancies between the networks reconstructed by these two approaches are expected. Both methodologies are easily generalizable to various biological systems and, in the context of infectious diseases, can deconvolve kinase-mediated signaling mechanisms in host cells or parasites. In the future, integration of TPS and TREKING will facilitate developing more informative network models. Integrating TPS and TREKING methodologies would allow 1) revealing the functionality of kinases and their inter-connections that alter cellular phenotypes along with their temporal enzymatic activity during changes of cellular phenotypes, and 2) linking functional non-kinase proteins that directly modulate cellular phenotypes to their upstream, functional kinase regulatory pathways that control the activity and function of the proteins.

## Methods

### Cell lines

Primary human brain microvascular endothelial cells (HBMECs; Cell Systems Cat# ACBRI 376) were cultured on rat tail collagen type I (5 μg/cm^2^; Corning Cat# 354236) in HBMEC culture media (Lonza Cat# CC-3202) at 37°C and 5% CO_2_. HBMECs were obtained at passage 3 and used until passage 9.

### Lysate preparation

HBMECs were seeded in 6-well plates (Corning Cat# 353046) at 55,000 cells/well and grown for 4 days with media change every other day. On the day of lysate collection, cells were equilibrated in serum-free culture media for 1 hour and then treated with thrombin at a final concentration of 5 nM for 5, 15, 30, 60, 120, 180, 240, 360 minutes. After the indicated incubation periods, cells were washed twice with ice-cold phosphate buffered saline (PBS) and lysed in sodium dodecyl sulfate (SDS) lysis buffer (50 mM Tris-HCl, 2% SDS, 5% glycerol, 5 mM ethylenediaminetetraacetic acid, 1 mM sodium fluoride, 10 mM β-glycerophosphate, 1 mM phenylmethylsulfonyl fluoride, 1 mM sodium orthovanadate, 1 mM dithiothreitol, supplemented with a cocktail of protease inhibitors (Roche Cat# 4693159001) and phosphatase inhibitors (Sigma-Aldrich Cat# P5726)). Cell lysates were clarified in filter plates (Pall Cat# 8075) at 2,671×*g* for 30 minutes, after which they were stored at −80°C until use. Cell lysates from three biological replicates were collected.

### Western blot

All the gel electrophoresis was performed using Bolt^™^ 4–12% Bis-Tris mini protein gels. Proteins were transferred to PVDF membranes using iBlot 2 (Thermo Fisher Scientific Cat# IB21001) or iBlot 3 (Thermo Fisher Scientific Cat# A56727) dry blotting system. Primary antibodies were used at concentrations recommended by the manufacturer (see Table S1 for antibody information). Antibody to GAPDH (Cell Signaling Technology Cat# 97166, RRID AB_2756824) was used as loading control at 1:2000 dilution. Blots were imaged using Bio-Rad ChemiDoc imaging system and signals were quantified using ImageJ2 (https://imagej.nih.gov/ij/, version 2.3.0). Background correction was done for each band by subtracting background signals nearby the band. The signals from proteins of interest were first normalized to the signals from GAPDH, and then the signals at each time point were normalized to the signals at time zero for the fold change of phosphorylation from basal level. Phosphorylation on activation sites of 25 kinases and 3 non-kinase proteins were evaluated at nine time points (0, 5, 15, 30, 60, 120, 180, 240 and 360 min after thrombin treatment). Western blot was performed on cell lysates collected from three biological replicates.

### Reconstruction of TPS network

The input time series data for TPS were collected from western blot experiments. A portion of the data were previously published ^13^. The input network, an undirected subnetwork of the PPI network that connects the phosphorylated proteins to the source node(s), was generated using the Omics Integrator implementation of the Prize-Collecting Steiner Forest algorithm ^17,18^. Time series phosphorylation data, protein prizes and interactome network were required for generating the input network. Protein prizes were obtained using significance scores for each time point relative to the first time point and to the previous time point for each profile. The interactome network was compiled by combining human PPIs from iRefIndex ^28^ (https://irefindex.vib.be//) and kinase-substrate phosphorylation interactions from PhosphoSitePlus ^29^ (https://www.phosphosite.org). Omics Integrator was run with dummy edge weight of 10, edge reliability of 10, degree penalty of 0, and 100 randomize prize runs. Significance scores for TPS run were computed with paired Student’s t-tests comparing the phosphorylation intensity at each time point and the first time point (“firstscores”) or comparing the phosphorylation intensity at the current time point and the preceding time point (“prevscores”). Threshold for significance scores was set to 0.05, above which measurements are considered non-significant. Proteinase-activated receptor 1 (F2R/PAR1) was designated as the sole source node for TPS run, as it is the predominant thrombin receptor and the only member of PARs mediating thrombin-induced phosphoregulation in endothelial cells ^30,31^. An optional partial model was provided for TPS run, which is a directed, signed kinase-substrate phosphorylation interaction network from PhosphoSitePlus ^29^ (https://www.phosphosite.org).

### Visualization

**Fig. 2.**
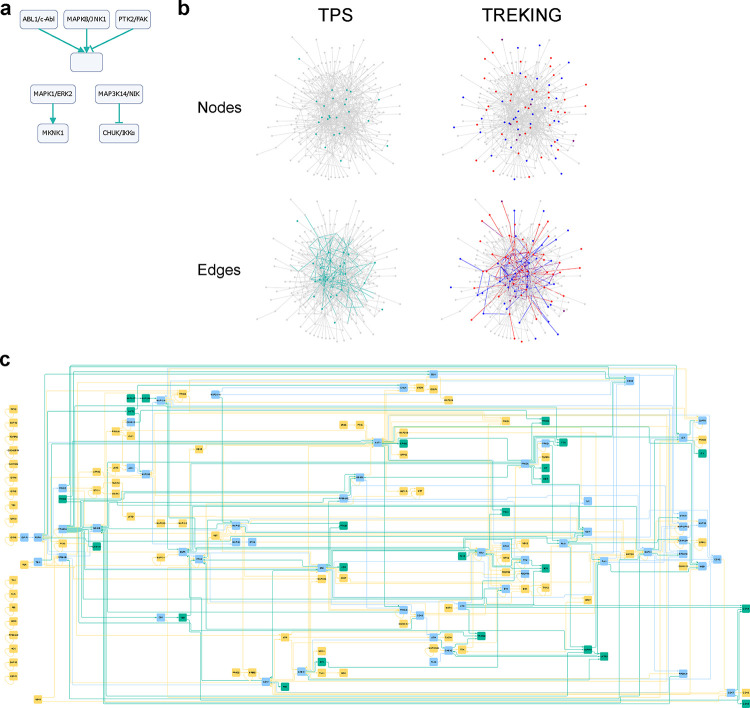
TPS infers common and unique kinases and signaling cascades compared to TREKING models. **a** Signed edges in the summary network generated by TPS. **b** Comparison of kinase phosphosignaling networks built by TPS and TREKING. The background network in gray includes all kinase-kinase phosphorylation interactions from the kinase-substrate phosphorylation database PhosphoSitePlus. Turquoise nodes are the kinases with time series phosphorylation data used to inform TPS network; turquoise edges are the kinase-kinase phosphorylation interactions inferred by TPS algorithm. Blue and red nodes are the kinases predicted by tKiR to have only barrier-weakening and barrier-strengthening functionality, respectively; purple nodes are the switch kinases having both barrier-weakening and barrier-strengthening functionalities but at different time frames. Blue and red edges are the interactions predicted by TREKING that are associated with only barrier-weakening and barrier-strengthening activity, respectively; purple edges are the interactions associated with both barrier-weakening and barrier-strengthening activities. **c** The combined kinase signaling network inferred by TPS and TREKING methodologies. Kinases and kinase-kinase interactions inferred by TPS or TREKING only are in turquoise or moccasin, respectively; kinases and kinase-kinase interactions inferred by both TPS and TREKING are in sky blue.

Phosphosignaling networks in Fig. 2b were visualized using NetworkX (https://github.com/networkx/networkx), a Python package for analyzing complex network structures. Phosphosignaling networks in other figure panels were visualized using Cytoscape (https://cytoscape.org, version 3.9.1), with layouts generated from yFiles layout algorithms (https://www.yworks.com/products/yfiles-layout-algorithms-for-cytoscape).

## Supplementary Material

Supplement 1 **Table S1. Antibody information and western blot results on the subset of proteins used to inform TPS network in this study.** This is a Microsoft Excel workbook containing 13 spreadsheets with antibody information and densitometry results for the 11 protein antibody targets.

Supplement 2 **Table S2. The undirected subnetwork generated by the Omics Integrator implementation of the Prize-Collecting Steiner Forest algorithm.** This is a Microsoft Excel workbook containing one spreadsheet.

Supplement 3 **Table S3. The summary network generated by the TPS algorithm.** The edge types are: A – ProteinA activates ProteinB; I – ProteinA inhibits ProteinB; N – ProteinA regulates ProteinB but the edge sign is unknown. Undirected edges are removed from the summary network. This is a Microsoft Excel workbook containing one spreadsheet.

Supplement 4 **Table S4. The kinase-kinase edges in the summary network generated by the TPS algorithm.** The edge types are: A – ProteinA activates ProteinB; I – ProteinA inhibits ProteinB; N – ProteinA regulates ProteinB but the edge sign is unknown. Undirected edges are removed from the summary network. This is a Microsoft Excel workbook containing one spreadsheet.

## Figures and Tables

**Fig. 1.**
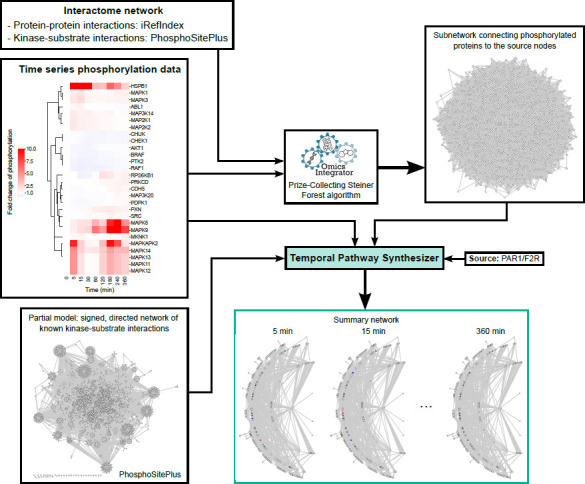
Workflow for reconstructing a kinase-mediated phosphosignaling network controlling brain endothelial barrier integrity after thrombin stimulation using TPS. HBMECs were treated with thrombin for 0, 5, 15, 30, 60, 120, 180, 240 or 360 min. Phosphorylation levels (at activation sites) of 28 proteins (25 kinases and 3 non-kinase proteins) were evaluated by western blot and quantified by fold change from the basal level (media only) after normalization to GAPDH. The interactome network was compiled by combining human PPIs from iRefIndex and kinase-substrate phosphorylation interactions from PhosphoSitePlus. An undirected subnetwork was generated using the Omics Integrator implementation of the Prize-Collecting Steiner Forest algorithm, incorporating the time series phosphorylation data and the interactome network. The subnetwork, kinase-substrate phosphorylation interactions, and time series phosphorylation data were input into the TPS algorithm, with PAR1/F2R as the source node. TPS outputs included 1) the kinase-mediated signaling network (directed and signed) that controls post-translational modifications in HBMECs after thrombin treatment, and 2) activity of each protein in the subnetwork at the corresponding time points.

**Table 1.** List of computational approaches for building and analyzing protein phosphosignaling networks.

Methodology	Description	Input experimental data	Output model	References
CausalPath	Extract causal links in pathways by analyzing changes in high-throughput proteomics profiles on the background of prior knowledge captured in biochemical reaction knowledgebases	High-throughput proteomics data	A set of causal links between measurable molecular features that can explain correlated changes in a given set of proteomics and other molecular profiles	Babur *et al*. ^4^
Dynamic Bayesian inference	Infer network structure and probabilistic relationships among a set of variables over time without user-set tuning parameters	Time-course phosphoprotein data	A graphic model consisting of kinase nodes and casual relationships between them with probability distribution function assigned	Hill *et al*. ^5^ Hill *et al*. ^6^ Merrell and Gitter ^7^
Information theory	Quantitatively predict strength of underlying complex relationships between proteins from noisy data based on the formalism of information theory	Single-cell protein phosphorylation data	A network denoted by the amount of mutual information transduced by the signaling pathways in response to external signals	Cheong *et al*. ^15^ Krishnaswamy *et al*. ^16^
Differential equations	Describe biochemical processes in the system using a set of coupled differential equations and predict temporal and spatial evolution of the latent variables	Kinetic parameters of biochemical reactions	A set of interacting molecular species trackable (amount and cellular localization) over time	Frohlich, Loos, and Hasenauer ^9^
Logic modeling	Model predictive signaling pathways based on networks of logic gates that specify how input signals lead to changes of cellular state	Functional biochemical data and phosphoproteom ics data	A data-optimized Boolean model (PPIs are in logical representations) that can predict cell responses to specific biological stimuli	Saez-Rodriguez *et al*. ^10^ Vaga *et al*. ^11^
KiRNet	Generate kinase-centered, functional network models by mapping kinase hits from functional kinase inhibitor screens onto a PPI network using network propagation approach	Phenotypic data from small-scale chemical screens	A focused subnetwork centered around the predicted key kinases that represents the proteins and relationships most critical for the phenotype of interest	Bello *et al*. ^12^
TREKING	Inform functionality of kinases and kinase signaling networks with temporal resolution based on temporal phenotypic readouts and existing biochemical data	Phenotypic data from small-scale chemical screens	A set of local kinase phosphosignaling networks describing the connections between functional kinases associated with temporal cellular phenotypes	Wei *et al*. ^13^
TPS	Assemble paths through which phosphosignals propagate from source node(s) based on the temporal activity of proteins and known PPIs	Temporal phosphoproteom ics data	A summary network that compiles the valid pathway models that depict how phosphosignals may propagate from source node(s)	Köksal *et al*. ^14^

## Data Availability

Code and a portion of the data used in this study have been published previously. The remaining data generated in this study is available in the supplementary information.
