# Supplementary material for: Systems-level reconstruction of kinase phosphosignaling networks regulating endothelial barrier integrity using temporal data
Source: bioRxiv. 2024 Oct 18:2024.08.01.606198. Originally published 2024 Aug 5. Preprint. [Version 2] doi: 10.1101/2024.08.01.606198 (PMC11326140; doi:10.1101/2024.08.01.606198)
Supplement: Supplement 6 [file media-6.pdf]

Supplementary Information

Systems-level reconstruction of kinase phosphosignaling networks regulating endothelial barrier integrity using temporal data

Ling Wei[1,*], John D. Aitchison[1,2,3], Alexis Kaushansky[1,2,4], and Fred D. Mast[1,2,*]

[1]Center for Global Infectious Disease Research, Seattle Children's Research Institute, Seattle, WA 98109, United States

[2]Department of Pediatrics, University of Washington, Seattle, WA 98105, United States

[3]Department of Biochemistry, University of Washington, Seattle, WA 98105, United States

[4]Department of Global Health, University of Washington, Seattle, WA 98105, United States

*Corresponding author. E-mail: ling.wei@seattlechildrens.org and fred.mast@seattlechildrens.org

This PDF file includes:

Legends for Supplementary Data S1 to S5

Other supplementary materials for this manuscript include the following:

Supplementary Data S1 to S5

Supplementary Data S1. Antibody information and western blot results on a subset of proteins used to inform TPS network in this study. This is a Microsoft Excel workbook containing 13 spreadsheets with antibody information and densitometry results for the 11 protein antibody targets (8 kinases, 3 non-kinases) evaluated in this study.

Supplementary Data S2. The undirected subnetwork generated by the Omics Integrator implementation of the Prize-Collecting Steiner Forest algorithm. This is a Microsoft Excel workbook containing one spreadsheet.

Supplementary Data S3. The summary network generated by the TPS algorithm. The edge types are: A – ProteinA activates ProteinB; I – ProteinA inhibits ProteinB; N – ProteinA regulates ProteinB but the edge sign is unknown. Undirected edges are removed from the summary network. This is a Microsoft Excel workbook containing one spreadsheet.

Supplementary Data S4. The kinase-kinase edges in the summary network generated by the TPS algorithm. The edge types are: A – ProteinA activates ProteinB; I – ProteinA inhibits ProteinB; N – ProteinA regulates ProteinB but the edge sign is unknown. Undirected edges are removed from the summary network. This is a Microsoft Excel workbook containing one spreadsheet.

Supplementary Data S5. Proteins and interactions involved in thrombin signaling through PARs. Information is from the Reactome pathway – thrombin signaling through proteinase activated receptors (PARs) (stable identifier: R-HSA-456926). This is a Microsoft Excel workbook containing two spreadsheets.
